# Endoscopic Endonasal Skull Base Surgery—state of the art

**DOI:** 10.3171/2020.4.FocusVid.Intro

**Published:** 2020-04-01

**Authors:** Juan C. Fernandez-Miranda, James J. Evans, Paul A. Gardner, Carl H. Snyderman, Pablo F. Recinos

**Affiliations:** 1 Department of Neurosurgery, Stanford University, Palo Alto, California;; 2 Department of Neurosurgery, Thomas Jefferson University Hospital, Philadelphia;; 3 Department of Neurosurgery, University of Pittsburgh Medical Center, Pittsburgh;; 4 Department of Otolaryngology, University of Pittsburgh School of Medicine, Pittsburgh, Pennsylvania; and; 5 Department of Neurosurgery, Cleveland Clinic, Cleveland, Ohio

It is with great enthusiasm that we present this collection of high-quality surgical videos for the evolving field of endoscopic skull base surgery. Video submissions from 13 skull base groups and 3 different continents have been selected based on their technical mastery, case complexity, or management of vascular complications. This collection represents the state of the art for the field of endonasal endoscopic surgery as we start a new decade that promises further advancements and innovations.

We are particularly thrilled to witness the worldwide expansion of expertise in this complex field, which has mainly resulted from a better understanding of the endonasal surgical anatomy and technique. This translation of knowledge and skill from the laboratory to the operating room is well illustrated here as expert surgeons demonstrate the compartments of the cavernous sinus, the transcavernous posterior clinoidectomy technique, the translacerum approach, various pituitary transposition techniques, the combination of open and endonasal approaches, the removal of giant and highly complex lesions considered inoperable by many, and even the clipping of intracranial aneurysms or the successful management of potentially devastating intraoperative vascular injuries.

There is no better way to assess the technical progression of this discipline than by evaluating the surgical videos of challenging cases as performed by master surgeons. These videos should serve as a reference for those who aspire to achieve mastery in this field, but the viewers and learners should not be fooled by the outstanding results presented here. These are the consequence of many years of training, dedication, and progressive experience. The learning curve for complex endonasal surgery is very steep, and there are no shortcuts. Intense laboratory work performing high-quality anatomical dissections, dedicated clinical fellowship training at high-volume skull base centers, and a lifelong commitment to excellence in endonasal endoscopic surgery are the essential ingredients to become an expert in the field.

